# Nanoenabled Bioelectrical Modulation

**DOI:** 10.1021/accountsmr.1c00132

**Published:** 2021-08-30

**Authors:** Aleksander Prominski, Pengju Li, Bernadette A. Miao, Bozhi Tian

**Affiliations:** † Department of Chemistry, The University of Chicago, Chicago, Illinois 60637, United States; ‡ The James Franck Institute, The University of Chicago, Chicago, Illinois 60637, United States; § The Institute for Biophysical Dynamics, The University of Chicago, Chicago, Illinois 60637, United States; ∥ Pritzker School of Molecular Engineering, The University of Chicago, Chicago, Illinois 60637, United States

## Abstract

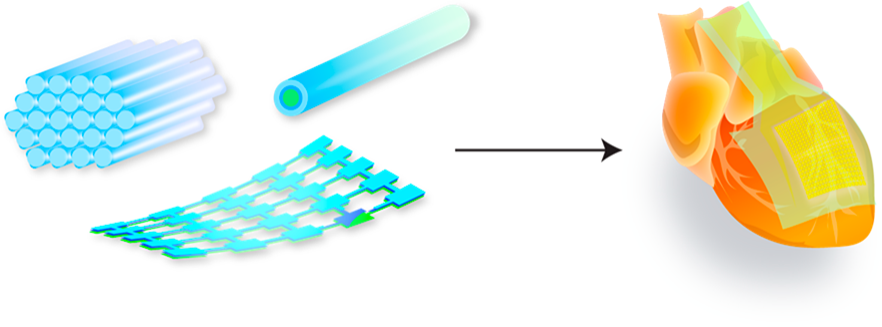

Studying the formation and interactions between biological systems and artificial materials is significant for probing complex biophysical behaviors and addressing challenging biomedical problems. Bioelectrical interfaces, especially nanostructure-based, have improved compatibility with cells and tissues and enabled new approaches to biological modulation. In particular, free-standing and remotely activated bioelectrical devices demonstrate potential for precise biophysical investigation and efficient clinical therapies. Interacting with single cells or organelles requires devices of sufficiently small size for high resolution probing. Nanoscale semiconductors, given their diverse functionalities, are promising device platforms for subcellular modulation. Tissue-level modulation requires additional consideration regarding the device’s mechanical compliance for either conformal contact with the tissue surface or seamless three-dimensional (3D) biointegration. Flexible or even open-framework designs are essential in such methods. For chronic organ integration, the highest level of biocompatibility is required for both the materials and device configurations. Additionally, a scalable and high-throughput design is necessary to simultaneously interact with many individual cells in the organ. The physical, chemical, and mechanical stabilities of devices for organ implantation may be improved by ensuring matching of mechanical behavior at biointerfaces, including passivation or resistance designs to mitigate physiological impacts, or incorporating self-healing or adaptative properties.

Recent research demonstrates principles of nanostructured material designs that can be used to improve biointerfaces. Nanoenabled extracellular interfaces were frequently used for either electrical or remote optical modulation of cells and tissues. In particular, methods are now available for designing and screening nanostructured silicon, especially chemical vapor deposition (CVD)-derived nanowires and two-dimensional (2D) nanostructured membranes, for biological modulation in vitro and in vivo. For intra- and intercellular biological modulation, semiconductor/cell composites have been created through the internalization of nanowires, and such cellular composites can even integrate with living tissues. This approach was demonstrated for both neuronal and cardiac modulation.

At a different front, laser-derived nanocrystalline semiconductors showed electrochemical and photoelectrochemical activities, and they were used to modulate cells and organs. Recently, self-assembly of nanoscale building blocks enabled fabrication of efficient monolithic carbon-based electrodes for in vitro stimulation of cardiomyocytes, ex vivo stimulation of retinas and hearts, and in vivo stimulation of sciatic nerves.

Future studies on nanoenabled bioelectrical modulation should focus on improving efficiency and stability of current and emerging technologies. New materials and devices can access new interrogation targets, such as subcellular structures, and possess more adaptable and responsive properties enabling seamless integration. Drawing inspiration from energy science and catalysis can help in such progress and open new avenues for biological modulation. The fundamental study of living bioelectronics could yield new cellular composites for diverse biological signaling control. In situ self-assembled biointerfaces are of special interest in this area as cell type targeting can be achieved.

## 1 Introduction

Designing efficient strategies for interfacing biological structures with artificial materials is a formidable challenge. Such biointerfaces are of critical importance to fundamental studies of biophysical interactions between cellular components and their environment and are a basis for recording and stimulation applications that are significant in biomedical fields, especially neuroscience research.^1^ Nanotechnology-driven materials research for biological modulation has presented a number of advantages over approaches utilizing classical bulk materials and devices. Primary benefits of reducing the size of a device’s active components include increased resolution with which stimulation or recording can be performed. Interacting with biological matter on the cellular and subcellular level allows for a high degree of specificity and fidelity in the biointerface, which is especially important for neural interfaces, such as retinal implants for vision restoration.^2^ Additionally, nanomaterials can be part of larger assemblies, where single nanostructures can act as separate transducers enabling high-throughput parallel communication,^3^ e.g., for the realization of efficient brain–machine interfaces. Another advantage of nanostructured materials is their improved mechanical compliance with biological tissue. Due to small dimensions and reduced bending stiffness, nanostructures elicit minimal immune responses in in vivo applications, creating stable interfaces suited for chronic implantation. Matching of mechanics and size allows cells to adapt freely to and seamlessly contact with nanostructured interfaces.^4^ Furthermore, when nanodevices are sufficiently small, they can be internalized by certain cell types, resulting in intracellularly integrated systems.^5^ Overall, nanostructures enable efficient and biocompatible means of biological modulation critical for future translational applications of such technologies.

Although bioelectrical interfaces are primarily concerned with recording and eliciting action potentials in electrically excitable cells,^6^ investigation of the effects of electric fields on growth, development, and migration of nonexcitable cells is a growing direction as well.^7^ Nanostructured materials have applications in classic bioelectronics as electrode materials, where surface area nanoengineering has greatly improved recording and stimulation performance,^8,9^ and single nanostructures have been used to record subcellular electronic signals.^10^ Another side of bioelectronics is the synthesis and application of free-standing bioelectronic devices. Such devices can stimulate cells using light-induced phenomena, such as photovoltaic, photoelectrochemical, or photothermal effects.^11^ Stimulation with free-standing nanostructures benefits from the remote signal transduction mechanism; i.e., there is no need to connect the electrode to the current or voltage source, and as such an application of photoresponsive nanostructures that can stimulate native cells presents an important alternative to optogenetics.^12^ While more work is required to improve the stability and efficiency of free-standing nanostructures for photomodulation and convenient delivery methods have to be devised, we believe that this category of bioelectrical devices can achieve translational importance in the near future.

**Figure 1:**
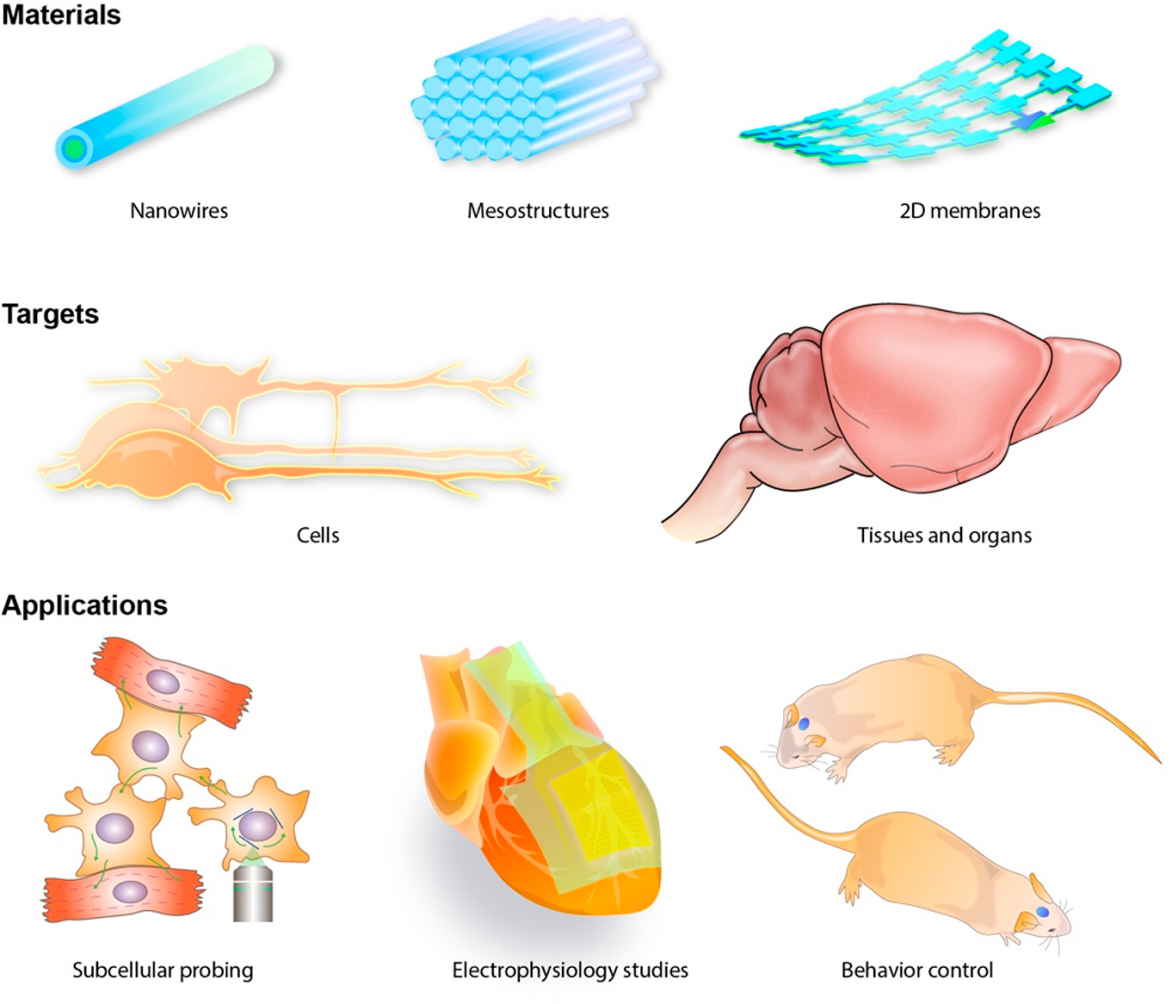
Materials engineering for stimulation of cells, tissues, and organs enables many important applications for biophysics research. Length scales of the modulation material can span through many orders of magnitude and surface nanostructuring improves compatibility with biological tissues and enables new functionalities, such as photoresponsive properties. Single free-standing nanostructures can be used to investigate cells on the subcellular level and study signal propagation pathways but can also be assembled into composite devices for tissue and organ-level studies of the electrophysiology or behaviors.

In this Account, we describe research and strategies for developing nanostructure-enabled bioelectrical interfaces for modulation of cells, tissues, and organs (Figure 1). We discuss the rationale, approaches, and challenges involved in design of modulation devices that interact with living systems on different levels of biological hierarchy. We follow with an analysis of recent developments focusing on materials synthesis strategies and design of modulation experiments that can influence a range of biological targets. Finally, we discuss our outlook on the future of nanoscale biomodulation in directions of subcellular biophysics, fully integrated bioelectronics, and intelligent, adaptable systems. We hope that our perspective on materials research for bioelectrical modulation will showcase the progress and promise of this important research direction and highlight the relevance of connecting materials design with characteristics of biological systems.

## 2 Modulation Across Different Length Scales

### 2.1 Cellular Scale

Biological systems extend across different length scales, making it necessary for bioelectrical devices to be designed based on their target applications. The choice of length scale, which spans from the nanometer to the meter, depends on the application and required resolution. Devices can be used for sensing and modulation in extracellular and intracellular studies and with cells, tissues, and organs. Sensing and modulation functions rely on signaling processes and can be related through electrical potentials often characterized by ion concentration and flow through cell membranes. Current research aims to improve sensing and modulation techniques through developing higher resolution and more biocompatible approaches.

Semiconductor nanomaterials can be used to achieve both nanoscale dimensions and high spatial resolution.^6^ Nanoscale semiconductor devices are promising developments for biomodulation at the cellular level due to their matched physical dimensions. Nanoscale semiconductors are obtained in various shapes and forms, including nanowires, mesostructures, and membranes (Figure 1). Silicon-based materials are of particular interest as they are inherently biocompatible and biodegradable compared to other semiconducting materials which might require cytotoxicity mitigation.

Although much progress has been made in bioelectronics, there still exist challenges in integrating devices with single cells. Difficulty arises with long-term biocompatibility, especially in devices based on bulky wired technologies. Therefore, research has focused on free-standing or self-assembly approaches that directly target cellular structures.

For single cell photothermal modulation, the process usually follows an optocapacitance mechanism.^13,14^ Since lipid bilayers can be treated as an electrical capacitor, their electrical capacitance can be modulated to control single cells. Short infrared light pulses can quickly change the membrane electrical capacitance by local heating, which depolarizes the plasma membrane and then elicits action potentials. The heating process increases the membrane electrical capacitance (*C*), which generates a capacitive current (*I*_*cap*_), following , where *Q* is the charge flow to/from the plasma membrane and *V* is the voltage across the membrane. Synthetic light absorbers such as gold nanoparticles and silicon nanowires^15,16^ make this process more convenient as visible and near-infrared light can now be used for stimulation.

Local heating can modulate membrane capacitance change and elicit action potentials at the single cell level. However, this capacitance change is temporary. Liu et al. showed that long-term membrane capacitance modulation is possible via genetically targeted chemical assembly of synthetic polymers directly over cell membranes.^17^ The authors observed decreased neuronal spiking when conducting polymers were deposited onto engineered neurons and increased spiking with insulating material production.^17^ This observation can be attributed to long-term membrane capacitance modulation. For example, when neurons are coated with a conducting polymer, electrical capacitance of the hybrid neuronal membrane increases due to large electric permittivity^18−20^ of the polymer, following *C* = ε_*o*_*ε*_*r*_*A*/*d*, where *A* and *d* are the surface area and the thickness of the capacitor, *ε_0_* is the vacuum permittivity and *ε*_r_ is the dielectric constant of the material. This capacitance change causes a decrease in spiking activity in the recorded single neurons.

Besides changing membrane electrical capacitance, local variation of electrochemical potential near a cell can yield single cell modulation. This variation can be achieved by either a direct electrochemical (i.e., wired-up device configuration) or photoelectrochemical (i.e., wireless device configuration) process. For example, nanoscale coaxial p-i-n silicon nanowires, upon forming extracellular interfaces, can serve as miniaturized photodiodes^4^ for photoelectrochemical elicitation of neural and cardiac action potentials.

### 2.2 Tissue and Organ Level

**Figure 2:**
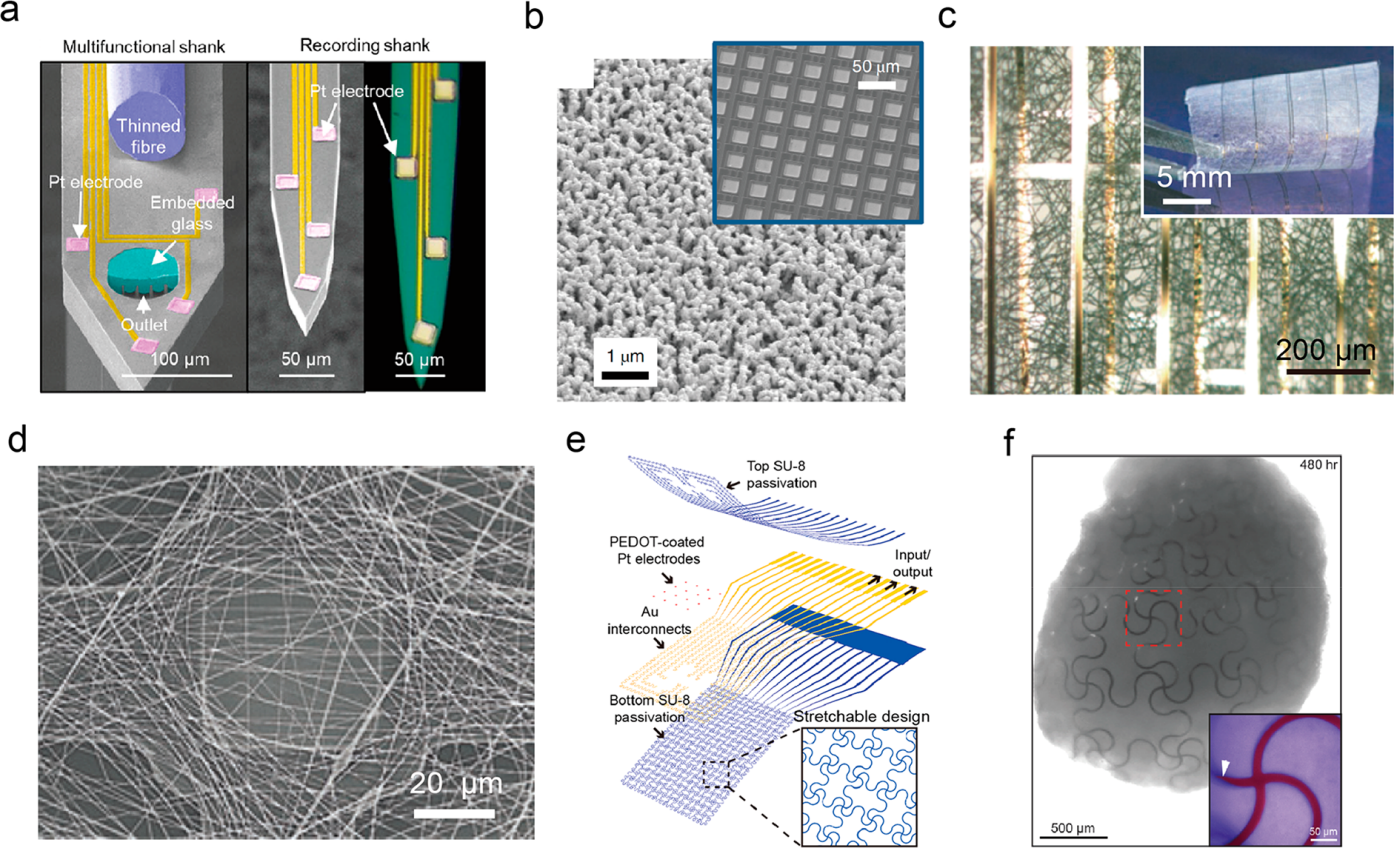
Recent developments in wired-up bioelectrical interfaces. (a) Multifunctional neural probes with electrical, optical, and microfluidic interrogation capability. Reproduced from ref (23). Copyright 2021 Springer Nature. (b) Modified porous platinum electrode surface with nanoscale bumps and gaps which were found to enhance cell adhesion and sealing. This surface modification method is applicable to large-scale multielectrode arrays (inset). Reproduced from ref (25). Copyright 2018 Springer Nature. (c) Biomaterial scaffolds with built-in electronics. Reproduced from ref (26). Copyright 2012 Springer Nature. (d) SEM image showing the stimulation/recording electrode was covered by a dense network of electrospun fibers as the scaffold for tissue regeneration and biological modulation. Reproduced from ref (27). Copyright 2016 Springer Nature. (e) Schematics of serpentine interconnects-enabled stretchable mesh MEAs and (f) its incorporation with an organoid. Reproduced from ref (28). Copyright 2019 American Chemical Society.

Compared with single cell modulation, materials for tissue- and organ-level stimulation usually require considerable mechanical flexibility to enable large contact areas for effective signal delivery. In addition, high-density and parallel configurations are required for multimodal modulation and recording with cellular resolution.^21,22^ Recent developments in fabrication of mechanically compliant nanobioelectronics with noninvasive soft tissue conformality and biomimetic integration (Figure 2) have shown to be promising for tissue- and organ-level stimulation research.

Bioelectronics with high-density device layouts could enable stimulation of dense systems, e.g., a neural probe with electrical, optical, and microfluidic stimulation/recording capabilities could be used to generally and holistically interface with the brain tissue.^23^ More importantly, miniaturizing electrode patterns to achieve cellular resolution could lead to simultaneous interrogation of a large number of cells spanning across the tissue, which allows signals to be delivered with high precision and quality. Early success of Utah microelectrode arrays (MEAs) directed research focus to multichannel neural stimulation and recording via penetrating probes. Initial designs used metal-tipped Si electrode arrays that allow only electrical interrogation.^24^ Recently, multimodal penetrating designs enabled incorporation of 4 Pt electrodes, 5 microfluidic channels, and an optical fiber in a single shank (Figure 2a).^23^

Many devices need to be surgically implanted into the biological matter, generating unwanted biological responses. Upon implantation, a layer of proteins is adsorbed onto the device, which can trigger a foreign body response by the immune system. In this immunogenic response, recruited macrophages release reactive oxygen species (ROS), which can degrade the implanted device. In addition, a significant increase in impedance is observed due to the capsulation of the device by fibrillar components. The device’s function deteriorates even further when it fails to synchronize with organ movements due to mechanical mismatch. Therefore, to achieve long-term physical and chemical stability, the following approaches can be considered: 1) utilizing soft and flexible device designs that are mechanically compliant with the surrounding tissue, 2) miniaturizing devices and modifying geometry to match those of surrounding cells, 3) adapting to macroporous designs for interpenetration of cellular networks, and 4) coating with biofluidic impermeable barriers to improve signal-to-noise ratio and long-term chemical stability. A well-established summary of biofluidic barrier materials was previously reported in the literature.^21^

Besides implantable devices, modulation biointerfaces can be more easily established in in vitro studies. While the microelectrode (∼10 μm in diameter) array with large-area integration is a widely accepted convention for performing tissue and organ interrogation with cellular-level resolution, benefits from introducing nanostructured surfaces are substantial. For example, nanoscale roughness and porosity (Figure 2b) may improve cell adhesion and charge injection capacity for future biointerfaces at the tissue or organ level. Tight sealing between nanopores and the cell membrane yields high signal-to-noise ratio in extracellular recording and even enables transient intracellular interrogation in cell networks and small tissues by optoacoustic poration.^25^ In addition, electrical and photoelectrochemical components may benefit from higher efficiencies due to increased electrode/electrolyte surface area and improved light absorption^9,29^ In the network of photoelectrodes, nanostructured components (i.e., nanowires) may potentially act as individual stimulators resolved by the laser spot size (∼500 nm), which would lead to even higher-resolution modulation.^30^ Benefits and applications of these nanoscale architectures will be discussed in detail in the next section.

For tissue or organ-level interfaces, biomimetic electrode designs promote seamless integration with tissues for stable and chronic bioelectric interface by minimizing the structural distinguishability of materials with surrounding cells and extracellular matrix (ECM). Nanoscale electrodes can be integrated with biomaterial scaffolds to achieve ECM-like designs, which enable cell seeding and tissue regeneration.^31^ Nanoelectronics built into flexible scaffolds can be used to probe physicochemical and biological microenvironments.^26^ Free-standing macroporous mesh-like electronics were made by interweaving Si nanowire field-effect transistors (FETs) and scaffold biomaterials, such as collagen, alginate, and PLGA. These materials were combined with nanoelectronics in the development of tissue cultures (Figure 2c). The hybrid scaffold provided successful seeding for neurons and cardiomyocytes, formation of a 3D engineered tissue, and real-time monitoring of local electrical activities, pharmacological responses, and pH changes. Later, Feiner et al. developed a hybrid cardiac patch which augmented stimulation functions into the tissue.^27^ As shown in Figure 2d, a highly porous bioelectronic scaffold was formed by integrating a porous electrode mesh with a nanofiber network, which facilitates tissue growth and interpenetration. With drug-loaded bioactive polymers capable of pharmacological interrogation, this scaffold has been shown to successfully record and regulate the pace of cardiac contraction. Recently, Li et al. reported free-standing mesh nanoelectronics compatible with organogenesis.^28^ During cell growth and reconfiguration, serpentine interconnects allow the mesh network to deform under cell–cell attraction forces into a 3D organoid (Figure 2e,f). This design highlights a promising direction in minimally invasive integration with tissues during organogenesis.

## 3 Nanomaterials Designs and Applications for Biological Modulation

### 3.1 Applications of Silicon Nanowires for Extracellular Biomodulation

A semiconductor material, silicon (Si) is used in bioelectrical devices because of its physical and chemical properties. Biocompatibility is of utmost importance as devices are interfaced with biological systems. Additionally, Si is a useful material because its biodegradability can be tuned to match the desired duration of functionality. While Si can be fabricated into a variety of configurations, SiNWs or Si nanomembranes are often used given their electrical and mechanical properties are suitable for a range of biointerfacing devices.^32−34^ Specifically, the highly tunable and controllable synthesis of SiNWs allowed them to be successfully implemented in various studies in our group.

In a work by Parameswaran et al., it was shown that optical neuromodulation could be conducted using free-standing coaxial p-type/intrinsic/n-type (p-i-n) SiNWs made of p-doped cores and n-doped shells.^4^ These SiNWs were used to wirelessly modulate primary rat dorsal root ganglion neurons through a photoelectrochemical process at the interface of neurons and SiNWs. Upon light stimulation, local depolarization of target neurons occurred with cathodic processes at the n-shell due to electron flow and movement of holes toward p-type Si core. Through this electron surface accumulation and photocathodic reaction, the plasma membrane was depolarized to elicit the action potentials. This development advances optical modulation methods as it is not mechanically invasive, nongenetic, and able to provide subcellular specificity.

**Figure 3:**
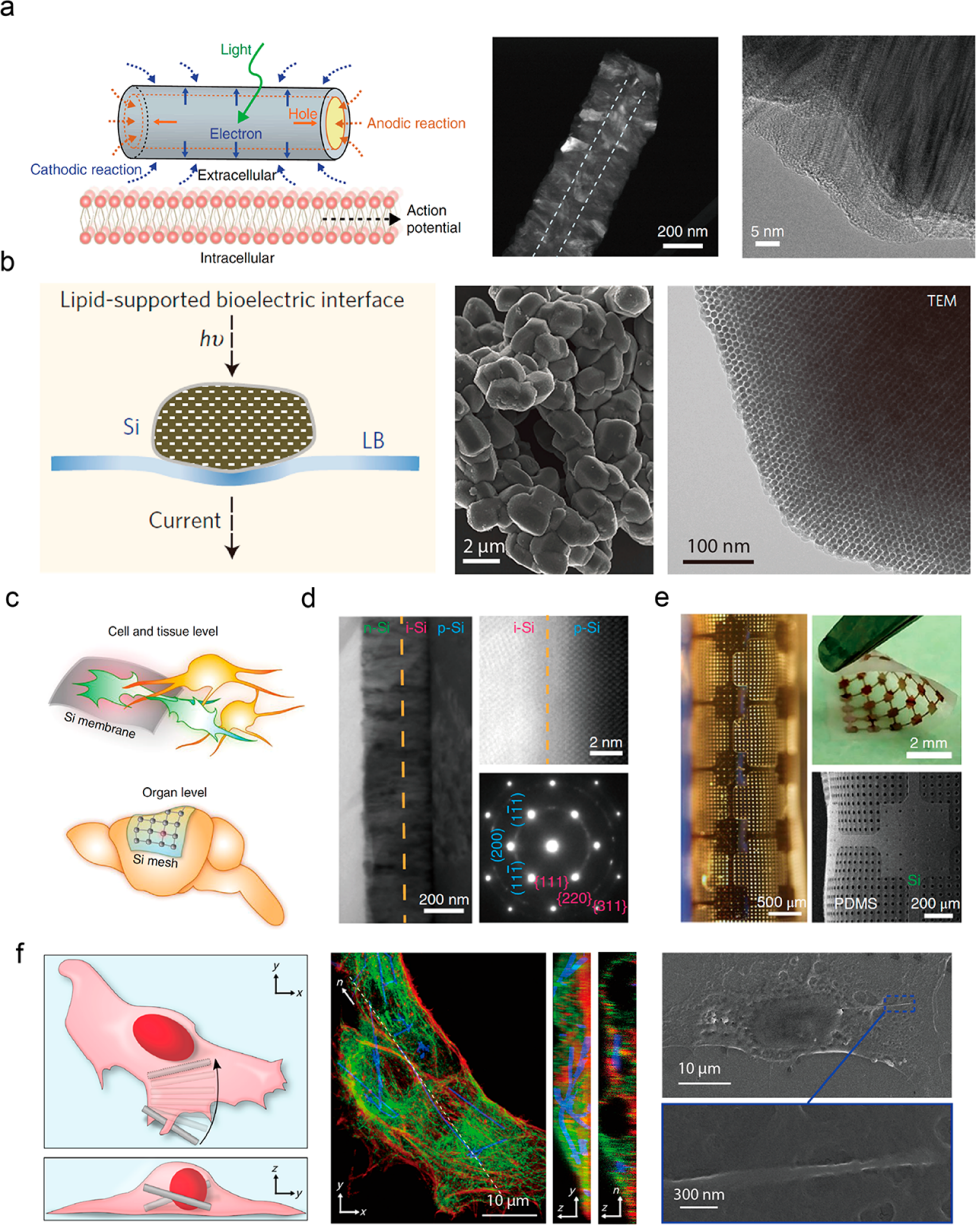
Silicon-based nanostructured materials for freestanding multiscale biological modulation. (a) Schematic of Faradaic currents generated by a p-i-n SiNW (left). HAADF-STEM (center) and TEM (right) images of a p-i-n SiNW. Reproduced from ref (4). Copyright 2018 Springer Nature. (b) Schematic illustration of the light-stimulated bioelectric interface of mesostructured Si (left). SEM (center) and TEM (right) images of mesostructured Si. Reproduced from ref (15). Copyright 2016 Springer Nature. (c) Schematics of Si structures for interfacing multiscale biological targets. In this selection, a multilayered p-i-n Si membrane was used for cell and tissue stimulation, and Si mesh was used for organ stimulation. (d) A cross-sectional TEM image (left) showing the p-i-n Si diode junction, a STEM image (upper right) showing the oxidation-free interface with a junction width of less than 1 nm, and a SAED pattern (lower right) indicating the nanocrystalline i-layer. (e) Optical micrograph (left), photograph (upper right), and SEM image (lower right) showing the Si mesh made of distributed holey Si membrane on porous PDMS substrate with exceptional flexibility. Reproduced from ref (29). Copyright 2018 Springer Nature. (f) Schematic illustration of internalization of SiNWs into the perinuclear region of the cell. (left) Confocal fluorescence micrograph and thin sections showing SiNWs internalization. (center) SEM micrograph of SiNW embedded under a cell membrane. (right) Reproduced from ref (36). Copyright 2016 AAAS.

This study also revealed and investigated the atomic gold present on nanowire surfaces. While the cores of the p-i-n-SiNWs were synthesized through a gold-catalyzed CVD growth, the Si shells were fabricated through vapor–solid deposition. Atomic gold species deposited on the surface during the synthesis were identified to have a beneficial effect on neuromodulation. Specifically, reduction of gold content in the p-i-n-SiNWs increased the energy needed for stimulation, suggesting that Au promotes the reaction at the interface by lowering the energy barrier for photoelectrochemical current generation (Figure 3a).^4^

Another work by Parameswaran et al. applied internalized SiNWs to cardiac systems,^30^ highlighting the promise of expanding the use of SiNWs as a noninvasive, nongenetic modulation technique. Modulation of cardiomyocytes through coordinated contraction provides possible means to treat heart disorders. This approach presents an alternative to electronic pacemakers and optogenetic cardiac modulation, which also uses light to control cardiac modulation but requires introducing photoresponsive ion channels into the cell membrane.

Using a free-standing composite mesh of a random SiNW network on polymer grid substrates and a moving laser input, neonatal rat cardiomyocytes and adult rat hearts ex vivo were stimulated to beat at targeted frequencies. The flexible composite mesh was produced with a polymer support of SU-8 and coaxial p-i-n-SiNWs. In contrast to stimulation processes in which each individual pulse induces one beat, this process involved periodically exposing target cells to optical pulsing until a targeted frequency was achieved. In this stimulation process, it was also investigated if beating frequencies could be maintained after removing the light stimulus. It was found that after increasing the break time in between stimulation periods to 10 min, target cardiomyocytes could no longer be stimulated, which suggests a “memory” mechanism within cells and that extended breaks in pacing return the culture to its initial state. Additionally, cytotoxicity tests revealed no significant effects of SiNWs on the health of cells and tissues. While this study presents a method for specific, noninvasive stimulation, it is currently limited to ex vivo studies as only visible light was used in this work.^30^ This can possibly be resolved with the use of near-infrared (NIR) light stimuli for small animals such as zebrafish or implantable light sources for large animals. As Si has a bandgap that allows light absorption over a range that includes NIR light, the use of NIR stimuli is a feasible approach to achieve light-enabled modulation in vivo.^35^

Optical modulation with SiNW can be advanced through rational design of mesoscale and nanoscale features. A work by Fang et al. demonstrated that surface topography modification that forms textured, porous SiNWs improved the photothermal effect compared with smooth SiNWs and could be used both intracellularly and intercellularly to modulate calcium dynamics.^16^

In this work, a strategic synthesis introducing porosity with periodic Au deposits was shown to improve the photothermal effect. Through periodic pressure perturbations in Au-catalyzed intrinsic SiNW (i-SiNW) growth and metal-assisted chemical etching (MACE), textured SiNWs with three-dimensional porous interiors were synthesized. Another advantage of these textured i-SiNWs is that rough surfaces may help form better interfaces than those with smooth SiNWs. These i-SiNWs were applied to mammalian and disease-related cell types, including the nonexcitable cancer cell line U2OS, which highlights the potential of expanding nanowire-based photostimulation to other biological applications. Additionally, this approach is scalable as the location of the Au nanoparticles that determines the porous structure can be modified with pressure modulation during batch synthesis.

### 3.2 Mesostructured Si Particles for Extracellular Biomodulation

Unconventional semiconducting meso- and nanostructures have been shown to form seamless interfaces with cells for biological modulation. Jiang et al. developed a three-dimensional heterogeneous mesostructured Si particle composed of hexagonally packed SiNWs (Figure 3b) by casting of a nanoscale template of SBA-15, CVD growth, and wet etching.^15^ The modulus of mesostructured Si was 2 orders of magnitude less than that of a bulk Si. In addition, the average Young’s modulus of the material further decreased after immersion in PBS solution, which could be attributed to deposition of gel-like degradation products on its surface. After formation of an interface between mesostructured Si and a cell membrane, remote photothermal stimulation could be achieved through rapid localized heating (∼1 °C ms^–1^) using 532 nm laser pulses. A brief increase in temperature raises the electrical capacitance of the membrane and activates ionic channels, eliciting action potentials. This optical stimulation method can generate spike trains in dorsal root ganglia neurons. Categorization of results revealed unnatural patterns, including alternating action potentials and subthreshold depolarization.^15^

### 3.3 Multilayered Si Membranes for Multiscale Biomodulation

Research on nanostructured semiconductor biointerfaces has been advanced by rational design considerations to match properties of multiscale biological systems. Freestanding semiconductor membranes are found to be promising candidates for tissue- and organ-level modulation because of their size-matching configuration and flexibility (Figure 3c). Jiang et al. reported a CVD-derived p-i-n Si membrane diode with a nanostructured crystalline i- and n-layer (∼140 and ∼190 nm in thickness, respectively) grown onto a single-crystalline p-layer (∼2 μm in thickness) used for extracellular and tissue stimulation (Figure 3d).^9^ The nanocrystalline layer design, which is reminiscent of that in Si thin-film solar cells, achieved the capacitive current up to 7400 pA due to enhanced light absorption and more efficient charge separation. In addition, the nanocrystalline i-/n-layer was found to exhibit natural roughness that enhances cell focal adhesion through which photocurrents can be delivered to cells and ex vivo tissue slices seamlessly and noninvasively. Ex vivo photoelectrical modulation using distributed Si membranes interfaced with brain slices of mice demonstrated excitatory postsynaptic current generation.

In the distributed p-i-n Si membrane, surface modification with Au nanoparticles led to further increase in capacitive and Faradaic currents (∼86 nA and ∼2 nA, respectively).^9^ These strong stimuli were sufficient for organ-level modulation. In addition, as shown in Figure 3e, the ultrathin distributed Si mesh (∼2.3 μm in thickness) is laminated on porous PDMS (∼120 μm in thickness) for stress dissipation, enabling a compliant and conformal interface with the soft and curvilinear brain cortex through van der Waals interactions. Illumination of the mesh induces enhanced neural activities where neural response rate is positively correlated with illumination power, indicating that Au-decorated Si mesh is a precise neuromodulator. Furthermore, animal behavior modulation at the forelimb primary motor cortex and triggered flexion-relaxion motion of corresponding limbs of an anaesthetized mouse have been used as a proof-of-concept application of the mesh.

### 3.4 Silicon Nanowires for Intra- and Intercellular Biomodulation

In addition to research focusing on substrate-bound SiNWs, incorporation of SiNWs into the cell body has been explored, as bulky substrates do not utilize inherent nanoscale properties of SiNWs. Substrate-independent designs can be achieved with internalization of SiNWs, which improves biocompatibility, makes single-cell devices possible, and advances current biomodulation developments.

In a work by Zimmerman et al., it was shown that high-aspect-ratio and label-free SiNWs were internalized into various cellular systems by endogenous phagocytosis through formation of encapsulation vesicles (Figure 3f).^36^ This demonstrates the ability to internalize SiNWs in a drug-like fashion that is inherently biocompatible as it is a substrate-free approach. It was noted that clustering of SiNWs occurred in the perinuclear region, particularly in vesicles and cytosol, highlighting the potential of targeting specific organelles and cellular areas. It was also shown that internalization can be cell-type specific as SiNWs were internalized in human umbilical vascular endothelial cells (HUVECs) and human aortic smooth muscle cells (HASMCs) but rejected by primary cardiomyocytes and dorsal root ganglia (DRG) neurons.

**Figure 4:**
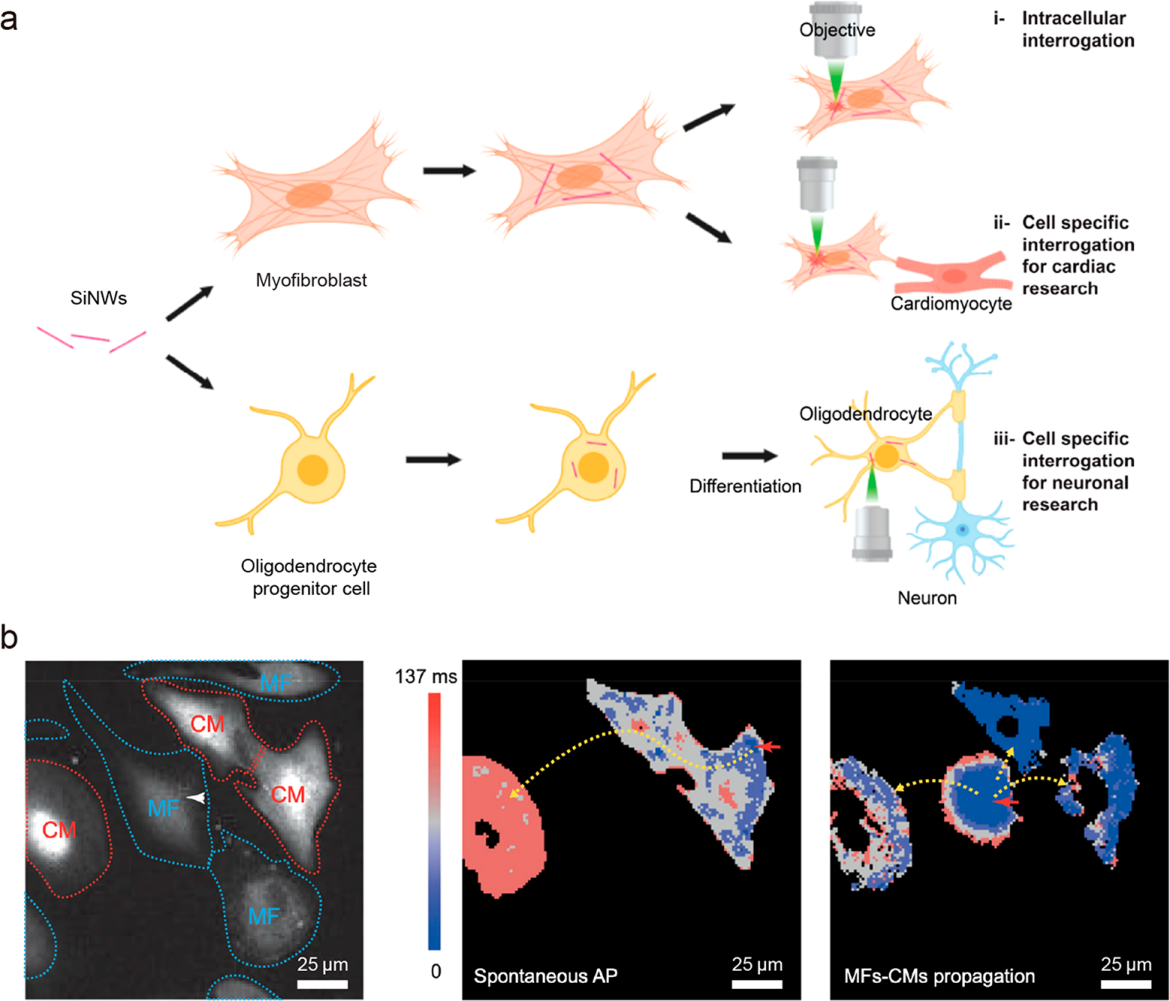
Silicon nanowires for intra- and intercellular biological modulation. a) Schematic illustration of creation of hybrid cells and their application for intra- and intercellular interrogations. Reproduced from ref (37). Copyright 2020 American Chemical Society. b) Fluorescent image (left) of myofibroblast-silicon nanowire hybrids (MF) in co-culture with cardiomyocytes (CM). Heat maps of calcium transient propagation in the case of spontaneous action potential (AP) (middle) and AP stimulated through hybrid MF (right). Reproduced from ref (5). Copyright 2019 PNAS.

In further studies, living bioelectronics were investigated in search of new methods of intra- and intercellular interrogation. Building on previous observations about internalization of SiNWs, hybrids of CVD-derived SiNWs and glial cells,^9^ myofibroblasts,^5^ and oligodendrocytes^37^ were obtained. With standard microscope laser setups (Figure 4a), it was possible to stimulate those cells with subcellular resolution and to enable neural or cardiac modulation in a remote region.^37^ Stimulation of multiple nanowires internalized within the same cell allowed for initialization of calcium transients and study of their propagation dynamics^5^ demonstrating a simple method of remote electrical stimulation of internal cellular structures without a complicated process or genetic modification.

Hybrid cells retain almost complete viability and continue to multiply, grow, and adapt to their environment. Therefore, in a co-culture, hybrid cells form intracellular junctions with other compatible cells. Co-culture of hybrids with other cell types, especially those that do not undergo nanowire internalization, allows for investigation of dynamics of cell–cell communication. For example, Jiang et al. used nanocrystalline Si nanowires internalized in glial cells as remotely controlled stimulators for neuromodulation.^9^ Upon light stimulation, localized heating triggered subcellular events, such as reactive oxygen species generation and organelle membrane depolarization/perforation modulating intra- and intercellular calcium ion flux dynamics. In addition, the photoacoustic effect accompanying the photothermal effect has been validated by repulsion of microtubules surrounding SiNWs, indicating the promise of remote biomechanical manipulation of subcellular structures.

This approach also enables proxy stimulation of cardiomyocytes through myofibroblast activation and neurons through oligodendrocytes (Figure 4a). Investigation of conduction velocity and calcium propagation dynamics between myofibroblasts and cardiomyocytes revealed the difference between conduction of spontaneous and stimulated action potentials (Figure 4b).^5^ Moreover, in vivo studies showed that hybrid cells were able to integrate successfully and electrically couple with native tissues. While studied hybrids are not yet efficient enough to overcome native compound action potential to allow pacing of an entire organ, such demonstration highlights the promise of engineering living bioelectronics using nongenetic approaches for clinical applications, such as treatment of arrhythmias and other diseases related to electrical decoupling of the myocardium.

### 3.5 Synthesis of Nanostructured Bioelectrical Interfaces from an Elastomer

**Figure 5:**
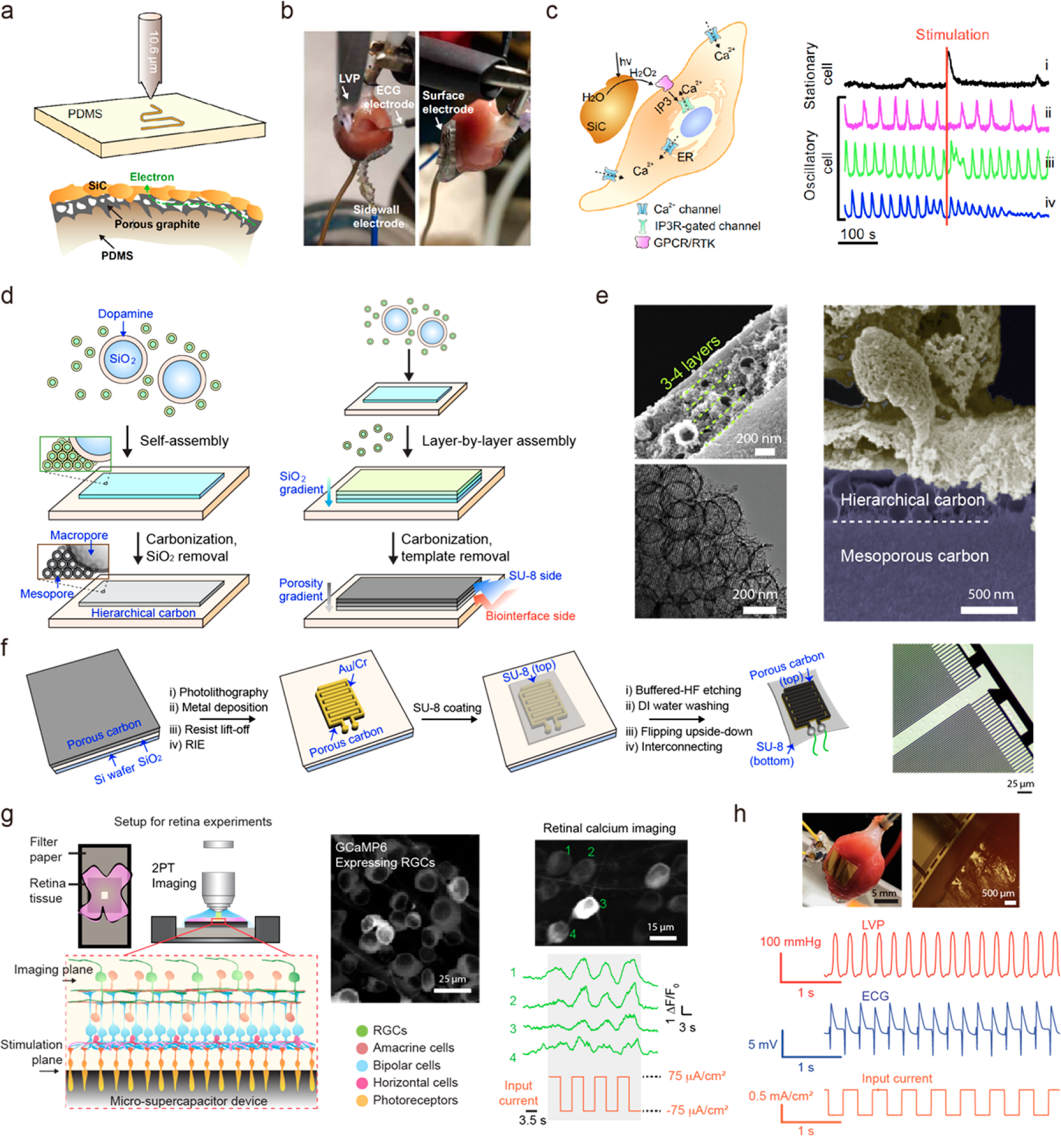
Synthesis of carbon-based nanostructured electronics for biological modulation. (a) Laser-ablation of PDMS turns the surface of PDMS into a SiC/porous graphite structure. (b) Photographs showing sidewall and surface electrode interfacing with an isolated rat heart for cardiac pacing. (c) H_2_O_2_ stimulation pathway (left) and fluorescent response of stimulated smooth muscle cells. (right). (d) A schematic of layer-by-layer assembly in the synthesis of hierarchical carbon membranes. (e) SEM of hierarchical carbon (left) and cross-sectional SEM of the biointerface formed between the material and cardiomyocytes. (f) A schematic of the fabrication approach for device patterning. (g) Stimulation of laminar retinal tissues. (h) Heart pacing setup (top) and physiology measurement during electrical pacing (bottom). (a)–(c) are reproduced from ref (41). Copyright 2020 AAAS. (d)–(h) are adapted from ref (9). Copyright 2021 Springer Nature.

Mechanically compliant bioelectronics usually require deposition of conducting or semiconducting materials onto soft polymers through drop-casting, filler mixing, transfer printing, or 3D printing. However, recently laser-assisted fabrication has become a competitive strategy due to its simple experimental setup and ease of implementation, which could potentially enable roll-to-roll production of large area, in-plane energy devices, microfluidics, and bioelectronics. CO_2_ laser ablation has been utilized to directly “write” porous or fibrillar graphene patterns on a variety of substrates, including polyimides, elastomers, papers, and types of wood, enabled by photothermal effects that accompany highly localized temperature and pressure.^38^ Adopting this strategy, Lin et al. patterned polyimide films with interdigited porous graphene electrodes as microsupercapacitors, showing specific capacitances of >4 mF/cm^2^ and power densities of ∼9 mW/cm^2^.^39^ Yang et al. reported an entirely laser-engraved wearable sensor system for sweat analysis. In addition to engraved microfluidic channels on flexible multilayers, laser-induced porous graphene on polyimide was used for multifunctional active components, including electrochemical sensors for uric acid and tyrosine detection as well as resistive sensors for monitoring temperature and strain.^40^ In our group, Nair et al. demonstrated laser ablation-assisted synthesis of semiconductor patterns on PDMS with submillimeter resolution (Figure 5a, top).^41^ In a nitrogen-rich atmosphere, thermal gradients converted the surface of PDMS into nitrogen-doped cubic silicon carbide (3C-SiC) with a nanoporous graphite bilayer structure underneath (Figure 5a, bottom) which was found to enhance capacitive properties. The resulting semiconductor–conductor junction enables fast prototyping of stimulation devices through direct writing and the ability to expand from planar structures to 3D forms. The mechanically compliant electrode has been found to be able to perform cardiac pacing in an ex vivo isolated heart by electrical stimulation (Figure 5b). Furthermore, beyond all previously reported laser-induced electrodes, this work first demonstrated photoelectrochemical modulation using the semiconductor 3C-SiC layer. Upon optical excitation, 3C-SiC exhibited photoanodic current generation, indicating oxidation of water to H_2_O_2_, which was found to elicit modulation of smooth muscle cells via regulation of inositol triphosphate receptors (IP3R) (Figure 5c). This photoelectrochemical system can potentially serve as a method for 1) therapeutic low-dose H_2_O_2_ delivery in the cell culture interface validated by calcium signal measurement and imaging, and 2) antibiotic lethal-dose H_2_O_2_ modulation with MnO_2_-enhanced photocurrent generation.

### 3.6 Self-Assembly of Nanoscale Building Blocks for Cell and Tissue Interfaces

Nanostructure engineering allows for the design of surfaces and volumetric materials with properties optimized for their application in biointerfaces in a similar fashion to how they are applied in energy science. While silicon-based materials have led to many advances in bioelectrical interfaces, developments involving other materials have shown to be promising. A recent study by Fang et al. shows that self-assembly of nanoscale building blocks allows hierarchical assembly of porous carbon membranes with improved mechanical and morphological compliance for applications in bioelectronics devices.^9^ Layer-by-layer assembly utilizing nanoscale micelles and dopamine-coated macroporous SiO_2_ enabled the synthesis of hierarchical meso- and macroporous (∼7 nm and 200–300 nm of pore size, respectively) carbon material (Figure 5d,e) which could be patterned to form a high-density microsupercapacitor-like stimulation device by lithography (Figure 5f). The device showed efficient electrical and biological coupling with cells, tissues, and organs due to mechanical compliance and the nanostructured surface. Specifically, this flexible system was used to record and stimulate cardiac systems and control electrophysiological parameters of isolated hearts, retinal tissues, and sciatic nerves. Additionally, carbon material shows purely capacitive operation, which minimizes risk of damage from undesired Faradaic reactions that can occur at the biointerface in the case of metal-based electrodes. The monolithic design of the device allowed binder-free fabrication of the electronic layers, which reduces risk of unwanted material release, and cytotoxicity studies showed no negative impact of the implants over cells and tissues.

The advantage of efficient material and interdigitated design comes from the presence of a confined electric field. Such a confined field is beneficial when working with monolayer cultures as it minimizes the energy necessary to elicit stimulation. It was shown that carbon-based devices successfully achieved overdrive pacing and subthreshold upregulation of rat cardiomyocytes in vitro. Another case in which the confined electric field is advantageous is in studying layered neural circuits, such as isolated retinas (Figure 5g). The device allowed for stimulation of “input neurons” (i.e., photoreceptors) and observation of the response of “output neurons”. It was shown that the intercellular signal could not propagate when communication pathways in tissue were inhibited pharmacologically which confirmed that the device stimulates only the most adjacent layer of cells. Finally, the device was used to pace isolated hearts ex vivo (Figure 5h) and stimulate sciatic nerves in vivo. Achieving energy-efficient stimulation demonstrates that nanostructuring of electrode material can have a beneficial effect on device performance in classic stimulation modes as well. Overall, this carbon-based membrane device presents an example of how energy science materials and device design paradigms can be successfully applied to enrich the repertoire of biomodulation techniques.

## 4 Outlook

Many important directions can be plotted for future research in nanoenabled bioelectronics. First and foremost, the efficiency of bioelectronic stimulation must be improved to minimize the energy required for stimulation and make such applications more resilient to sources of biological variability. Application of new material systems, such as a low-dimensional carbon-based composite,^42,43^ can potentially improve modulation efficiency. Advances in nanostructure synthesis and further modification on the nanoscale and atomic scale are promising to yield more robust signal transduction interfaces, where biomechanical sensing by the cellular machinery^44^ or biocatalysis^45^ may play additional roles. We believe that many paradigms previously successfully applied in energy science and catalysis can be adapted to improve bioelectronic devices. One recent example is in situ electrochemical generation of nitric oxide for neuronal signaling.^46^ We speculate that a similar approach should be achievable using photoelectrochemical effects. Moreover, utilization of nonconventional nanoenabled photonic processes such as upconversion^47^ and mechanoluminescence^48^ can enable new approaches to energy transduction in biomodulation.

Living bioelectronics is another direction that deserves particular attention due to significant benefits coming from seamless integration of natural and artificial elements. While this direction will benefit from improved nanostructure efficiency, there are concerns that require scientific insight. One of the most critical concerns is the stability of such hybrid cells and tissue. When pertaining to internalization of nanostructures, it must be determined how long the nanostructures will be active in the cells and how to control their persistence. One unanswered question is what happens to nanostructures when cells undergo mitosis. Keeping a large number of active hybrids in the cell population would be critical for long-term and chronic stimulation. Optimization of size, shape, and composition of nanostructures can enable control over their organization and location within the cytosol. The possibility of synthesis of bioelectronic structures in situ in living cells and tissues is a noteworthy idea. Recently, the conductive polymer polyaniline was synthesized directly onto cell membranes, which affected the conductivity of specific neuronal cells in vivo.^17^ While challenging, it would be extremely valuable to demonstrate assembly of photoresponsive materials or nanostructures within living cells. This approach has the potential to challenge optogenetic modulation approaches in terms of efficiency and safety.

While targeting single cells and subcellular regions with bioelectronics has been demonstrated, the next natural goal is to increase specificity of interrogation and study effects of stimulation on subcellular targets. Examples of such targets can include the cell membrane, organelles, cytoskeleton, liquid condensates, and extracellular vesicles. Such studies will bring important biophysical insight into the internal functionality of cellular machinery and enable new bioelectronic solutions. Modulating cells through triggered release of extracellular vesicles or through mechanical changes to the nucleolus may be important in future stimulation approaches. Subcellular insight will be used to further the design of nanostructures for internalization and creation of living bioelectronics as well.

Another frontier that we would like to highlight is creation of adaptable bioelectronics. Living systems are, in general, highly active environments undergoing constant change. We should think about designing or programing our materials to respond to presented conditions. Computer-assisted or intrinsically enabled closed-loop response systems are critical for realization of such adaptable materials. With increased complexity of autonomous behavior of nanostructured materials, we will be moving closer to achieving truly nanorobotic bioelectronic systems.

Finally, much as genomics and proteomics redefined how we study molecular and cellular biology, we envision that “*bioelectromics*”, if established through a suite of complementary characterizations and nanoenabled probing, can redefine our approach to electrophysiology, synthetic biology, and biomedical science. While efforts using traditional electrophysiology tools, combined with molecular biology and quantitative biophysical tools, have elucidated how single cells and tissues behave electrically, a systems-level understanding of bioelectrical activities and their heterogeneities at the subcellular level is lacking. Additionally, new synthetic biology or cellular engineering principles, especially those from nongenetic perspectives, would help establish autonomous, autoregulatory, cell-like materials. *Bioelectromics* may serve as a powerful bioengineering “blueprint” for completely new efforts in synthetic biology. Alongside chemical and transcriptional modules currently explored in the synthetic biology community, nanoenabled understanding of bioelectrical driving forces in cells and tissues may allow us to expand the diversity and application domains of cellular engineering.
